# Zinc and pH modulate the ability of insulin to inhibit aggregation of islet amyloid polypeptide

**DOI:** 10.1038/s42003-024-06388-y

**Published:** 2024-06-27

**Authors:** Samuel D. McCalpin, Lucie Khemtemourian, Saba Suladze, Magdalena I. Ivanova, Bernd Reif, Ayyalusamy Ramamoorthy

**Affiliations:** 1 Biophysics Program, University of Michigan, Arbor, MI 48109 USA; 2 Department of Chemistry, University of Michigan, Arbor, MI 48109 USA; 3 Institute of Chemistry and Biology of Membranes and Nanoobjects (CBMN), CNRS - UMR 5248, Institut Polytechnique Bordeaux, University of Bordeaux, 33600 Pessac, France; 4 Bayerisches NMR Zentrum (BNMRZ) at the Department of Biosciences, School of Natural Sciences, Technische Universität München, Munich, Germany; 5 Helmholtz-Zentrum München (HMGU), Deutsches Forschungszentrum für Gesundheit und Umwelt, Institute of Structural Biology (STB), Ingolstädter Landstr. 1, 85764 Neuherberg, Germany; 6 Department of Neurology, University of Michigan, Arbor, MI 48109 USA; 7 Michigan Neuroscience Institute, University of Michigan, Arbor, MI 48109 USA; 8 Biomedical Engineering, Macromolecular Science and Engineering, University of Michigan, Ann Arbor, MI 48109 USA; 9 National High Magnetic Field Laboratory, Department of Chemical and Biomedical Engineering, Institute of Molecular Biophysics, Neuroscience, Florida State University, Tallahassee, FL 32310 USA

**Keywords:** Molecular conformation, Biophysical chemistry

## Abstract

Aggregation of the human islet amyloid polypeptide (hIAPP) contributes to the development and progression of Type 2 Diabetes (T2D). hIAPP aggregates within a few hours at few micromolar concentration in vitro but exists at millimolar concentrations in vivo. Natively occurring inhibitors of hIAPP aggregation might therefore provide a model for drug design against amyloid formation associated with T2D. Here, we describe the combined ability of low pH, zinc, and insulin to inhibit hIAPP fibrillation. Insulin dose-dependently slows hIAPP aggregation near neutral pH but had less effect on the aggregation kinetics at acidic pH. We determine that insulin alters hIAPP aggregation in two manners. First, insulin diverts the aggregation pathway to large nonfibrillar aggregates with ThT-positive molecular structure, rather than to amyloid fibrils. Second, soluble insulin suppresses hIAPP dimer formation, which is an important early aggregation event. Further, we observe that zinc significantly modulates the inhibition of hIAPP aggregation by insulin. We hypothesize that this effect arose from controlling the oligomeric state of insulin and show that hIAPP interacts more strongly with monomeric than oligomeric insulin.

## Abstract (web summary)

Authors describe the combined ability of low pH, zinc, and insulin to inhibit fibrillation of islet amyloid peptide and find that insulin mediates this effect in two ways.

## Introduction

Type-2 Diabetes (T2D) has emerged as a major burden on international healthcare systems. In 2017, over 450 million individuals suffered from T2D, and global prevalence is expected to rise in the next decade^1,2^. The pathophysiology of T2D is defined by an inability to properly regulate blood glucose due to reduced insulin production and by an increased resistance of targeted tissues to secreted insulin. Insulin dyshomeostasis is believed to arise from inflammation, oxidative stress, ER stress, and amyloid stress, all of which are toxic to insulin-secreting pancreatic β-cells^3^. Amyloid stress has been particularly implicated in the pathogenesis of T2D based on several observations. Deposits of amyloid are typically present in pancreases affected by T2D, and the extent of amyloid formation has been correlated with decreased β-cell mass and increased β-cell apoptosis in diabetic individuals^4^. The primary protein component of these amyloid plaques is the human islet amyloid polypeptide (hIAPP, also called amylin)^5^.

hIAPP is a 37-residue (Fig. S1) hormone that is co-secreted with insulin in response to elevated blood glucose^6^. Due to its association with T2D, the aggregation behavior of hIAPP has been the subject of an impressive body of research. In vitro, human IAPP spontaneously forms amyloid fibrils within a few hours at sub-micromolar concentrations^7^. Some other species, such as rats, cows, and dogs, have variants of IAPP that do not spontaneously aggregate under the same conditions, and these animals also do not naturally develop T2D^6^. However, the disease can be induced in these organisms by transgenic modification to express the human variant of IAPP^8^. Consequently, hIAPP has become a popular research target for the development of therapies against T2D^9,10^. Notable anti-amyloid drug research candidates include polyphenol small molecules, peptides, antibodies, and lipid and metal nanoparticles^11–25^. Many such molecules and complexes inhibit hIAPP aggregation in vivo, but no drug currently exists for T2D which targets the amyloid plaques or toxic aggregation intermediates.

The physiological milieu offers a potential blueprint for discovering and designing effective therapeutics against hIAPP aggregation. Within pancreatic β-cells, hIAPP is stored in secretory granules, alongside zinc, insulin, and the C-peptide, at concentrations orders of magnitude higher than what has been observed to spontaneously form toxic aggregates in vitro^26–28^. Secretory granule lifetimes are on the order of 100 h or more, and little to no islet amyloid is observed in individuals without diabetes, so some aspect of its in vivo environment must act against toxic hIAPP aggregation^29^. Determining this factor and its mode of action on hIAPP aggregation could provide a model for the development of a drug with a similar mechanism of action. In pursuit of this goal, we describe here the combined effects on hIAPP aggregation and toxicity of three conditions and cofactors present in the secretory granule: acidic pH, Zn^2+^ ions, and insulin^28^.

Acidic pH, zinc, and insulin have been individually established as inhibitors of hIAPP aggregation^27,30–39^. The inhibitory effects of pH and zinc act via interactions involving residue H18 of hIAPP^31,34^. At low pH, His exists in its protonated form. Based on its proximity to the amyloidogenic core region of hIAPP, protonated H18 likely causes electrostatic repulsions between hIAPP molecules and thus disfavors their self-association. Protonation of the N-terminus likely also provides an additional electrostatic barrier to hIAPP self-association at low pH^40^. On the other hand, Zn^2+^ ions coordinate with histidine residues, likely inhibiting hIAPP aggregation by direct interactions with its lone histidine, H18. Our lab has previously described this interaction in detail^30–32^. At physiological pH, zinc reduced the extent of hIAPP fibril accumulation, with bimodal effects on the kinetics. Low concentrations of zinc delayed the onset of hIAPP aggregation and reduced amyloid fibril density, while high concentrations of zinc enhanced aggregation. Zinc only accelerated aggregation at acidic pH^30^. A model was developed in which zinc inhibits hIAPP aggregation by coordinating 4–6 hIAPP molecules via their H18 residues^31^.

Though all previous studies agree that insulin is a potent inhibitor of hIAPP, there is less agreement on the specific nature of the interaction^18,27,33,35–37,41–50^. A molecular mapping study by Gilead et al. determined that the hIAPP/Insulin interaction primarily involved residues 7–19 of hIAPP and residues 7–21 of the insulin B chain^51^. Most structural models derived from experimental and computational data agree that these regions of the peptides form the main interaction interface, but they broadly disagree on the overall structure of the complex and on the specific interactions that stabilize it^44–46,49,52–55^. For example, based on the structure of a crystallized homodimer of a maltose-binding protein-hIAPP construct, Wiltzius et al. proposed a model for a helical hIAPP-insulin heterodimer in which hydrophobic and aromatic interactions promote binding between helices in each peptide^44^. In contrast, Wei et al. characterized the interaction between insulin and rat IAPP by NMR and developed a structural model in which a helical heterodimer was stabilized by both hydrophobic interactions and salt bridges between the insulin B chain and the N-terminal region of rat IAPP^45^. While those models both describe helical subunits of hIAPP and insulin, several alternative models from molecular dynamics (MD) simulations suggest that hIAPP may instead interact with insulin by forming intermolecular β-sheets involving the same peptide regions in addition to the amyloidogenic core region of hIAPP (residues 20–30)^52,53,55^.

The situation is further complicated by considering that hIAPP and insulin might interact via aggregated or multimeric species of either peptide. Multiple studies have described insulin binding to preformed hIAPP fibrils, and one cross-linking experiment observed direct binding between multimeric forms of hIAPP and insulin^33,36,41,44^. Notably, insulin coordinates with Zn^2+^ ions to form crystalline hexamers in secretory granules and can form dimers and tetramers in the absence of zinc^56^. Data from MD simulations suggested that the oligomeric state of insulin governs its interaction with hIAPP^53^. Because the multimerization interface of insulin is the same region that binds to hIAPP, the monomeric form of insulin most efficiently interacted with hIAPP. However, there is no experimental evidence that corroborates this claim, and to the best of our knowledge, there are also no explicit experimental investigations of how the solution pH and the presence of zinc ions affect the interaction between hIAPP and insulin. The work presented here aims to address outstanding questions surrounding the hIAPP/insulin interaction regarding the oligomeric state of the species involved in the interaction, the effects of pH and zinc, and the structure of the hetero-complex.

## Results

### Insulin is a less efficient inhibitor of hIAPP aggregation at acidic pH

**Fig. 1:**
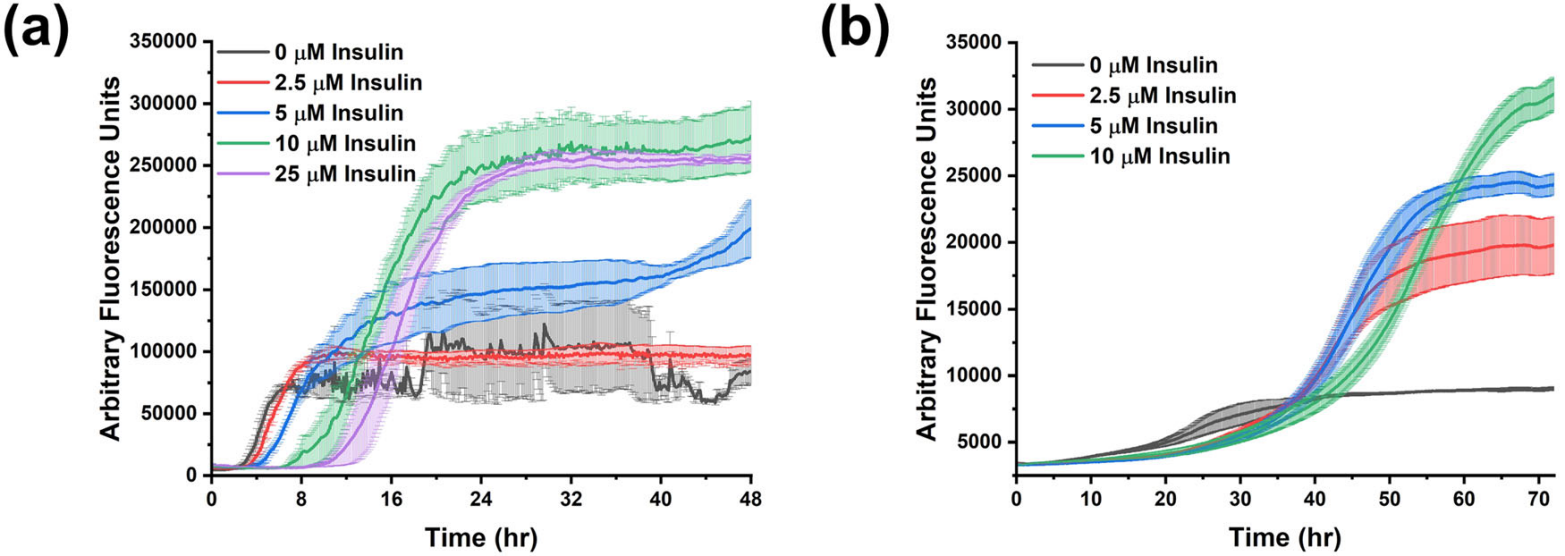
Thioflavin-T fluorescence assays with hIAPP and insulin. All samples were prepared with 5 µM hIAPP, 10 µM ThT, and 100 mM NaCl. Buffer conditions were **a** 10 mM Tris, pH 7.4 or **b** 10 mM NaAc, pH 5.5. Insulin controls contained 5 µM insulin. Normalized plots of these data can be found in Fig. S2.

The secretion pathway of hIAPP involves a change in pH from the secretory granule (pH 5.5) to the extracellular space (pH 7.4)^28^. Insulin is stored and secreted alongside hIAPP, and thus also present in both environments^57^. To determine whether insulin can effectively inhibit hIAPP aggregation in one or both pH conditions, we performed thioflavin-T (ThT) fluorescence assays at pH 7.4 and at pH 5.5 (Fig. 1). For both conditions, we also calculated the time to reach half of the maximum fluorescence (*t*_1/2_, Fig. S2). Under extracellular conditions (pH 7.4), hIAPP alone exhibited typical sigmoidal fluorescence kinetics, indicating the formation of amyloid fibrils within 10 h (Fig. 1a). In the presence of insulin, the lag time of the fibrillation increased with the concentration of insulin. A five-fold excess of insulin relative to hIAPP caused *t*_1/2_ to increase by ~4× (Fig. S2). These observations agreed with previous studies which found insulin to partially inhibit hIAPP aggregation in a dose-dependent manner^35,36,47^. We also observed a second increase in the ThT fluorescence intensity after 50 h for mixtures of hIAPP and insulin, but not for hIAPP alone (Fig. S3). The onset of the second fluorescence increase depended on the insulin concentration, being delayed by higher concentrations. To our knowledge, this behavior has not been previously reported for mixed hIAPP-insulin samples.

At low pH, the buffer conditions strongly influenced the fluorescence kinetics. In a MES buffer, the timescale of hIAPP aggregation was similar to that in pH 7.4 buffer (Fig. S4), while using a sodium acetate buffer resulted in much longer kinetics. Previous research mostly agrees that acidic pH slows hIAPP aggregation significantly, so it appears that MES likely interacts with hIAPP to accelerate its aggregation^33,34,38,39^. Accordingly, we used a sodium acetate buffer for all experiments at pH 5.5. In this condition the time course of hIAPP aggregation was longer, and insulin produced a smaller relative increase in the lag phase, a two times excess of insulin extending *t*_1/2_ by less than 2× (Fig. 1b). The second fluorescence increase also did not occur on the timescale of our experiment at the granule pH. Based on these results, the inhibitory effect of insulin on early-stage hIAPP aggregation was slightly less at acidic pH than at neutral pH. However, the later fluorescence increase was more strongly inhibited at pH 5.5, and the addition of insulin resulted in increased end fluorescence intensity in both pH conditions.

### hIAPP and insulin aggregate simultaneously into large, nonfibrillar aggregates

Two-step ThT fluorescence kinetics have been previously reported for several systems and arose primarily in two situations^58–66^. First, under certain conditions, some peptides formed aggregation intermediates, which induced a lower ThT fluorescence compared to the end aggregates. For example, Hasecke et al. observed distinct ThT fluorescence increases for both dimeric Aβ and hen egg-white lysozyme in which the first increase resulted from metastable oligomers and the second from end-stage fibrillar aggregates^62^. Insulin also has been demonstrated to cause multi-step ThT kinetics under certain conditions, such as high pressure, deriving from distinct conformational transitions^65^. The second scenario with multi-modal ThT fluorescence kinetics involved mixed peptide systems in which two peptides aggregated independently, and the two ThT fluorescence plateaus represented different timescales for the aggregation of each peptide. For example, Cukalevski et al. reported biphasic ThT kinetics for a binary mixture of Aβ40 and Aβ42 in which the Aβ42 formed fibrils during the first fluorescence increase, and Aβ40 aggregated during the second^66^. Given that insulin has exhibited two-step ThT kinetics from changes in aggregate morphology and that both hIAPP and insulin are known to form amyloid, it is reasonable to assume the two-step kinetics in Fig. S3 could represent either a change in aggregate morphology or sequential, independent aggregation of hIAPP and insulin.

**Fig. 2:**
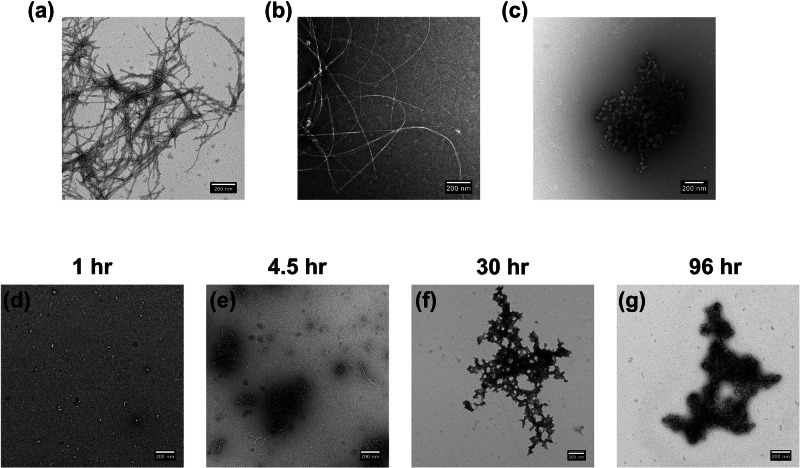
TEM images of IAPP and insulin aggregates. TEM micrographs of **a** 5 µM hIAPP, pH 7.4; **b** 5 µM hIAPP, pH 5.5; **c** 5 µM hIAPP, 10 µM insulin, pH 5.5; **d**–**g** 5 µM hIAPP, 10 µM insulin, pH 7.4. The pictures in panels a-c were collected after 96 hr sample incubation, while d-g were collected at the noted sample incubation times.

To determine whether a change in aggregate morphology occurred in the presence of both hIAPP and insulin, we performed negative-stain transmission electron microscopy (TEM) of a mixture of hIAPP and insulin over time. The TEM micrographs revealed that hIAPP alone formed typical fibrils at pH 7.4, but insulin caused the formation of large nonfibrillar aggregates (Fig. 2) in both pH conditions. Insulin that was incubated alone for 96 hr produced similar amorphous species to those in the mixture with hIAPP (Fig. S5), though the aggregates from the mixture were larger than any from insulin alone. Micrographs collected at several time points (Fig. 2d–g) showed that these aggregates began to form after 4 h and grew in size for approximately the next 24 h, until the second ThT fluorescence plateau. Formation of the nonfibrillar aggregates preceded the first increase in ThT fluorescence and there were no significant changes in aggregate morphology that corresponded with the second fluorescence increase and were observable by TEM. The ThT fluorescence increases may have corresponded to aggregate growth rather than distinct conformational or morphological changes, but we cannot make a definitive conclusion based on the TEM data.

**Fig. 3:**
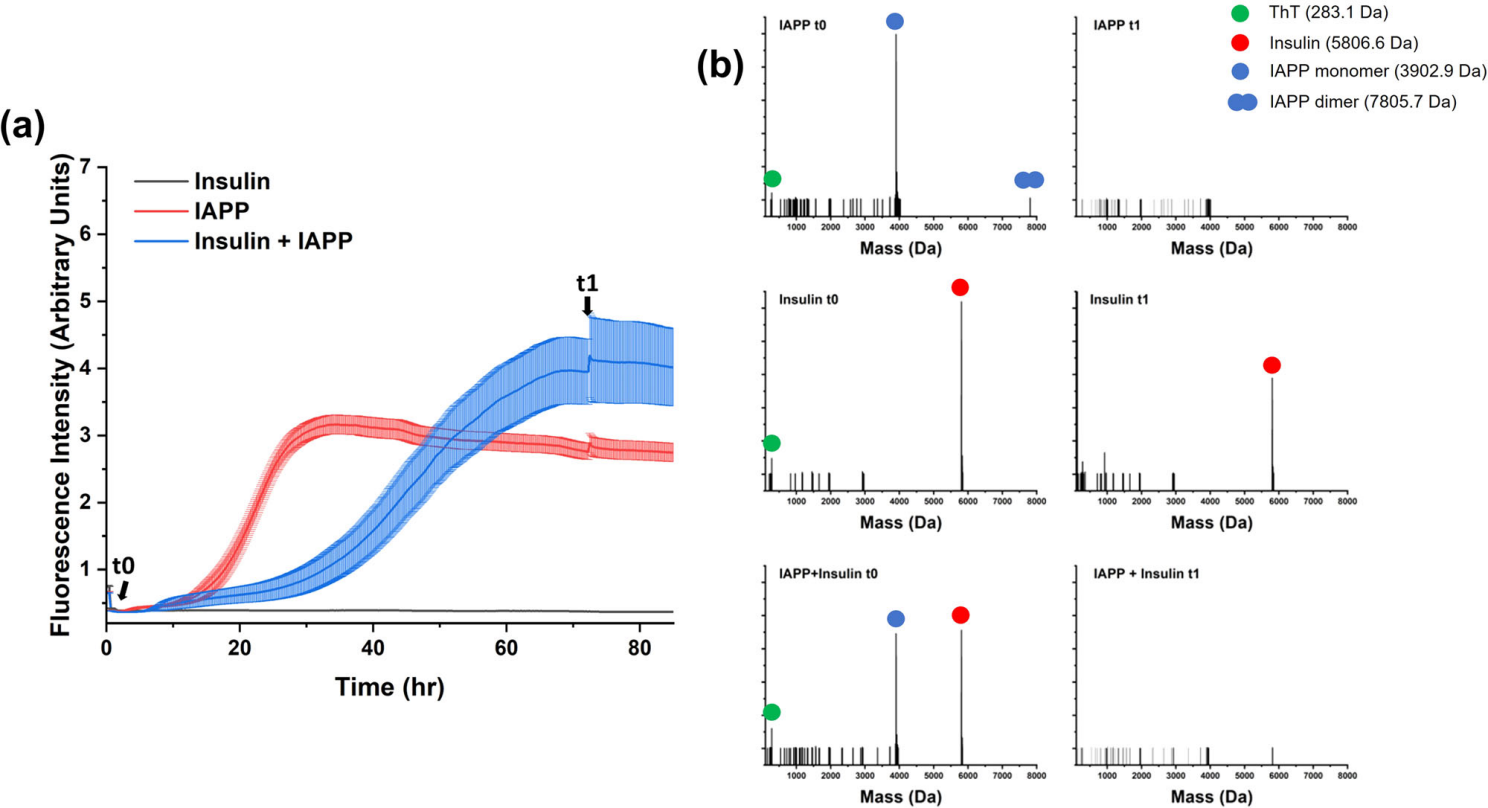
LC-MS indicates simultaneous aggregation of hIAPP and insulin. **a** hIAPP (5 µM) and insulin (5 µM) were mixed in pH 7.4 buffer (10 µM ThT, 10 mM Tris, 7 mM NaCl) and their aggregation was monitored by a ThT fluorescence assay. hIAPP and insulin controls were also prepared and monitored in the same way. **b** Aliquots from each sample were collected at the noted timepoints, centrifuged to remove insoluble material, and analyzed by LC-MS.

While the TEM micrographs revealed morphological details of the aggregates formed in the mixture of hIAPP and insulin, they could not determine whether aggregates contained one or both peptides or whether the peptides were sequestered by the aggregates sequentially or simultaneously. To elucidate these details, we performed an experiment based on tandem liquid chromatography-mass spectrometry (LC-MS). Aggregation reactions were begun and monitored by ThT fluorescence for a mixture of hIAPP and insulin and for controls with each peptide alone. Aliquots were taken at the initial time point and at the first ThT fluorescence plateau. These aliquots were centrifuged to remove large aggregates, and the remaining soluble species in the supernatant were characterized by LC-MS (Fig. 3). As expected, the MS data from the control samples showed a strong signal at the initial timepoint (*t*_0_) for masses corresponding to the appropriate peptide. After the first ThT fluorescence increase (*t*_1_), no signal remained from the hIAPP sample due to aggregation of hIAPP, while insulin alone maintained a strong signal from unaggregated, soluble species. For the hIAPP + insulin sample, both peptides were observed by MS at *t*_0_, but the signal from both peptides had disappeared at *t*_1_. Given that the nonfibrillar aggregate would have formed between *t*_0_ and *t*_1_, these results indicate that hIAPP and insulin simultaneously aggregated.

### Insulin weakly interacts with hIAPP to prevent its initial self-association and misfolding

**Fig. 4:**
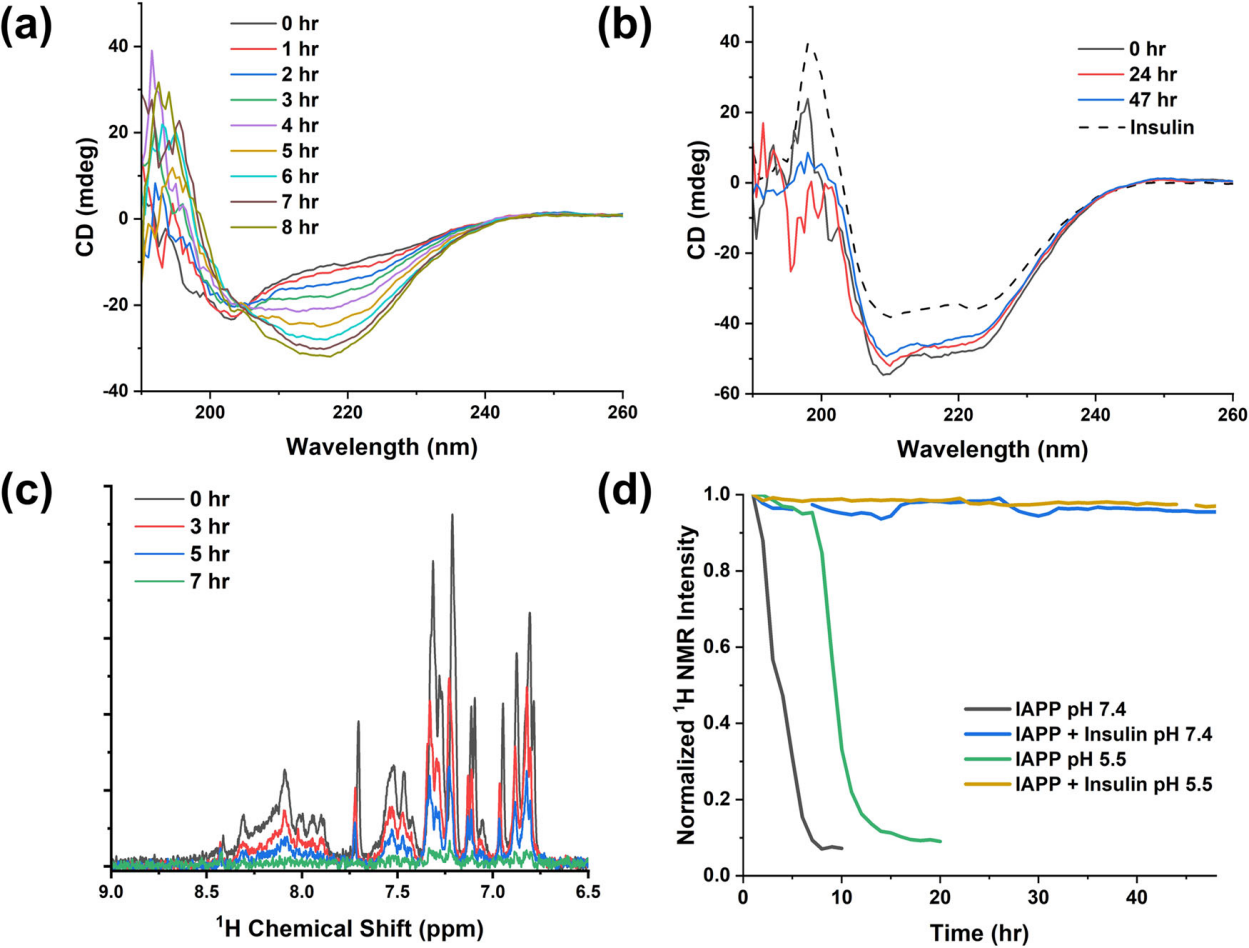
Insulin prevents early hIAPP aggregation events. **a** The CD spectrum of hIAPP was monitored over time and showed a transition from random-coil to beta-sheet after 8 h. **b** In the presence of insulin, the combined hIAPP and insulin CD spectrum did not change over time and appeared like the spectrum of insulin alone (dashed line). All CD samples were prepared with 25 µM hIAPP, 10 mM Tris, 100 mM NaCl, pH 7.4, and 25 µM insulin where applicable. **c** The ^1^H NMR spectrum of hIAPP at pH 7.4 decreased in intensity over time as the peptide aggregated. **d** Samples containing insulin did not lose intensity for at least 48 h. NMR spectra were collected using a 500 MHz spectrometer, and samples contained 50 µM hIAPP, 70 µM insulin where noted, and a pH 7.4 buffer (10 mM d_11_-Tris, 100 mM NaCl) or a pH 5.5 buffer (10 mM NaAc, 100 mM NaCl).

Electrospray ionization MS can preserve non-covalent protein complexes^67,68^. Large complexes or intermediates along the hIAPP and insulin aggregation pathways were not directly observed by our LC-MS experiments because they ionize less efficiently, but a soluble species with a molecular weight of 7805.7 Da, consistent with an oxidized non-covalently bound hIAPP homodimer, was observed for the sample of hIAPP alone (Fig. 3a). Dimerization of hIAPP is considered a crucial early step in the aggregation pathway of hIAPP, so it is significant to see this species in the MS data^44,69–72^. The hIAPP homodimer was not present in the hIAPP + insulin sample, indicating that insulin interferes with the initial self-association of hIAPP. Circular dichroism (CD) and NMR spectroscopy corroborated the effect of insulin on early events in the aggregation pathway of hIAPP (Fig. 4).

To monitor structural changes associated with early-stage aggregation events, we collected CD spectra at regular time intervals for samples of hIAPP and insulin. For hIAPP alone, the CD spectra displayed a strong negative minimum at 203 nm which shifted to 218 nm within 8 h, typical of the random-coil to β-sheet transition that occurs during amyloid formation (Fig. 4a). However, the spectra of hIAPP and insulin together showed negative minima near 209 nm and 222 nm, like the spectra of insulin alone, which did not change after 48 h (Fig. 4b). We could not deconvolute the spectral contributions of hIAPP and insulin, so we could not determine specific secondary structures in each peptide, but no β-sheet formation was observed over time. Similarly, we measured ^1^H NMR signal intensity over time and compared the aggregation kinetics of samples of hIAPP and insulin. Large, soluble species, such as amyloid fibrils and high-order oligomers, tumble slowly relative to the NMR timescale and exhibit broad NMR signals that are undetectable by typical solution NMR experiments. Accordingly, the ^1^H NMR spectra of the hIAPP alone sample decreased in intensity as hIAPP aggregated. The signal decayed entirely within 7 h at pH 7.4, and within 15 h at pH 5.5. Regardless, the addition of insulin nearly totally prevented the loss of the ^1^H NMR signal for at least 48 h in both pH conditions, consistent with an inhibition of early-stage aggregation events.

**Fig. 5:**
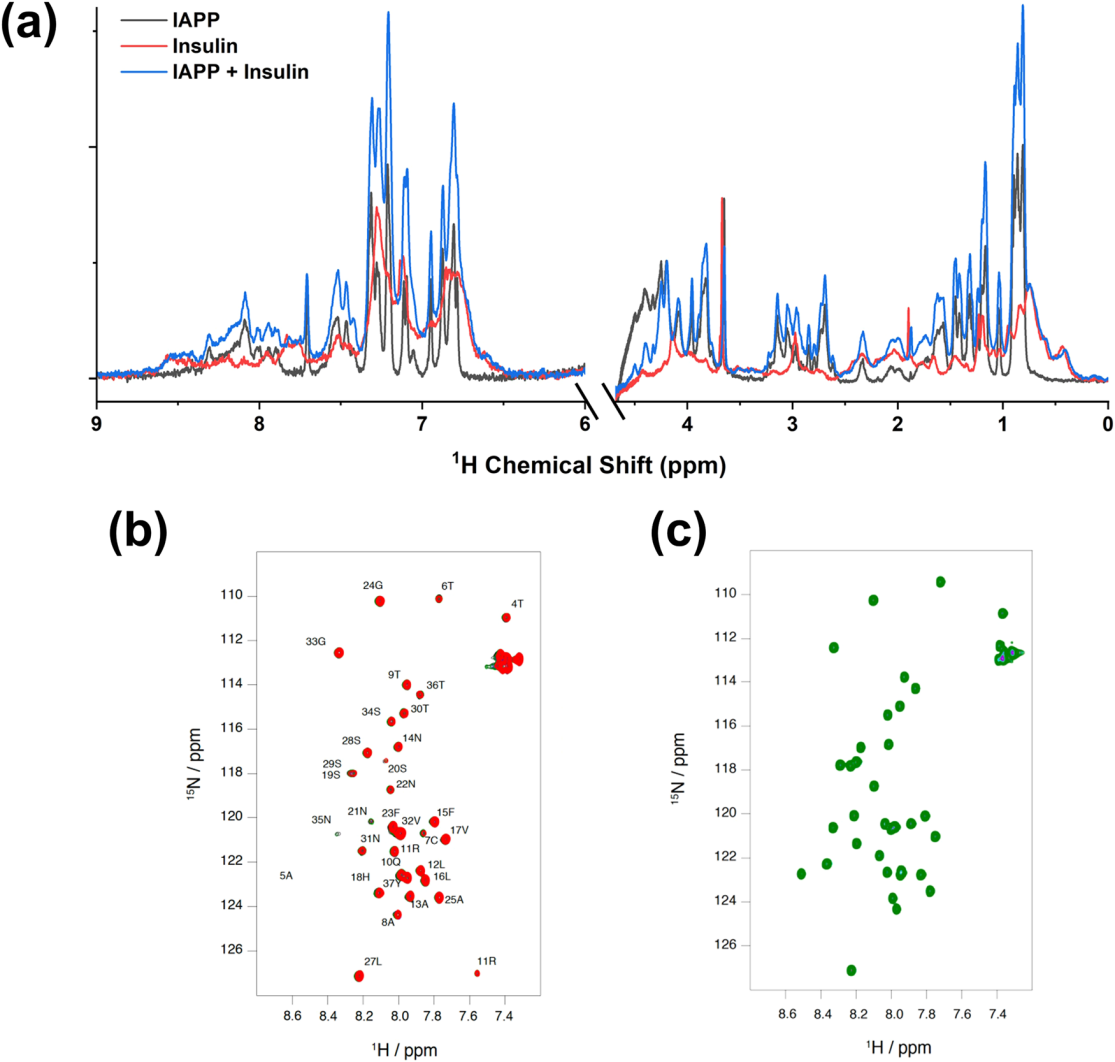
Probing interactions between hIAPP and insulin by NMR. **a** Peaks in the ^1^H NMR spectrum of mixed hIAPP and insulin appeared to be the sum of the spectra of hIAPP and insulin alone. Overlaid ^1^H-^15^N HSQC spectra of mixtures of 50 µM ^15^N-labeled hIAPP and 0 (black), 25 (gray), 50 (blue), 100 (green), or 200 (red) µM unlabeled insulin at **b** pH 7.4 and **c** pH 5.5 showed no discernible changes in chemical shifts in the presence of insulin. Resonances in **b** are labeled with hIAPP residue assignments. 1D NMR sample conditions are the same as in Fig. 4.

Despite strong evidence for an interaction between hIAPP and insulin, comparisons of ^1^H NMR spectra of hIAPP revealed no changes in chemical shifts upon the addition of insulin (Fig. 5a). Summing the spectra of hIAPP and insulin control samples produced a nearly identical lineshape to that of the spectrum of their mixture, with only small differences in peak intensities. ^1^H NMR spectra have an inherently low resolution of overlapping peaks, so we also collected 2D ^1^H-^15^N HSQC spectra of ^15^N-labeled hIAPP during titration with unlabeled insulin (Fig. 5b, c). However, chemical shift perturbations (CSPs) were negligible even after adding a four-fold excess of insulin. This was true for titrations performed at both pH 7.4 and pH 5. Based on models of helical IAPP-insulin heterodimers, we hypothesized that a helix-inducing solvent might increase the strength of the interaction by increasing the helicity of hIAPP and, thus, also the CSPs measured by NMR. With this in mind, we performed another titration of hIAPP with insulin in a 30% HFIP solvent, which is known to induce helical structure in hIAPP^73^, and collected the same set of HSQC spectra (Fig. S6), but the CSPs did not increase in magnitude.

### The ability of insulin to interact with hIAPP is dependent on its oligomeric state that is governed by the concentrations of zinc and insulin

**Fig. 6:**
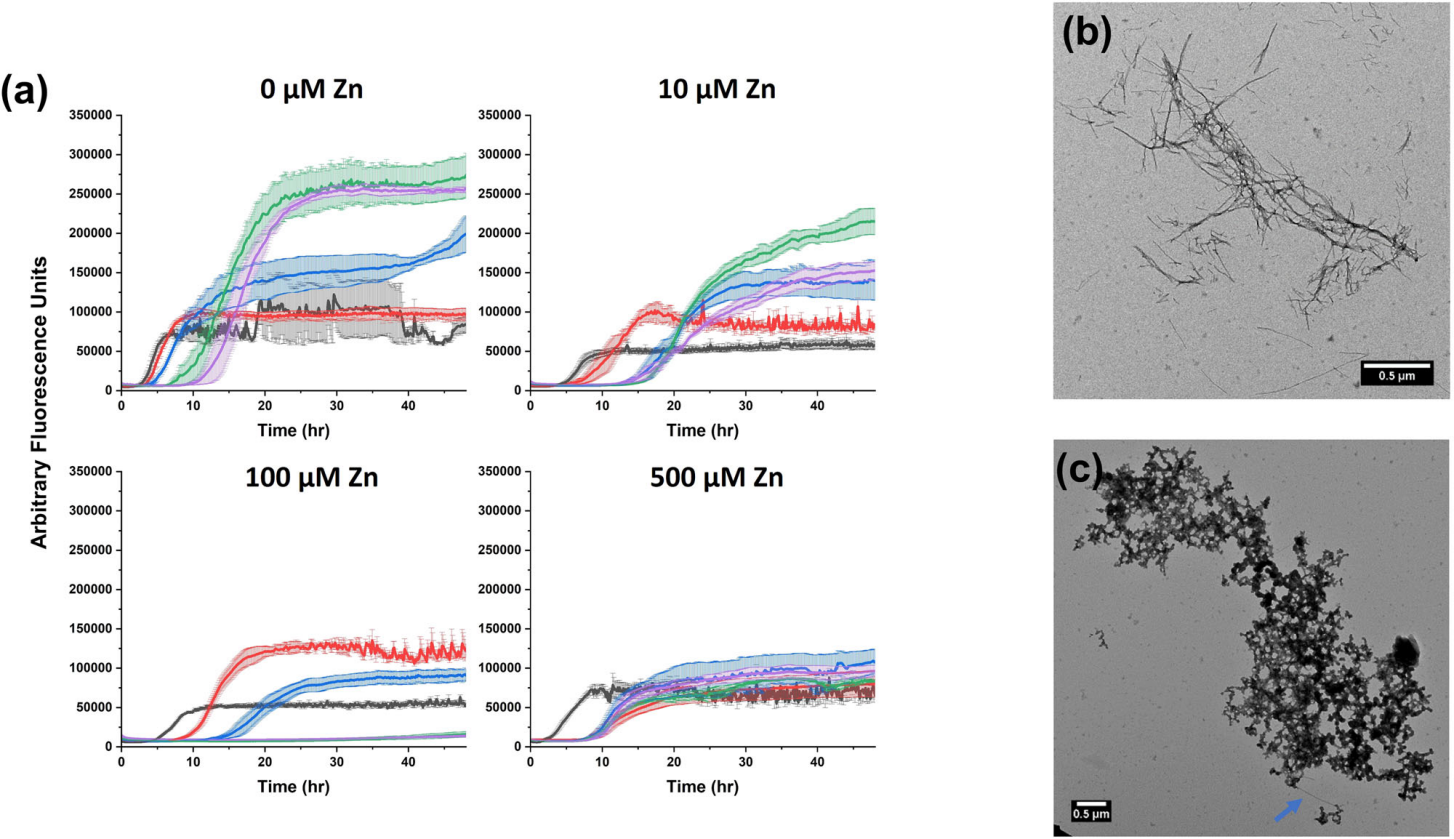
Aggregation of hIAPP in the presence of both zinc and insulin at pH 7.4. **a** ThT fluorescence assays were performed with 5 µM hIAPP, 0/2.5/5/10/25 µM insulin (black/red/blue/green/purple), the noted concentrations of ZnCl_2_, 10 µM ThT, 10 mM Tris, 100 mM NaCl, pH 7.4 samples. TEM micrographs were collected for samples with **b** 5 µM hIAPP, 0 µM insulin, and 5 mM ZnCl_2_ or **c** 5 µM hIAPP, 10 µM insulin, and 5 mM ZnCl_2_ after 96 h sample incubation.

**Fig. 7:**
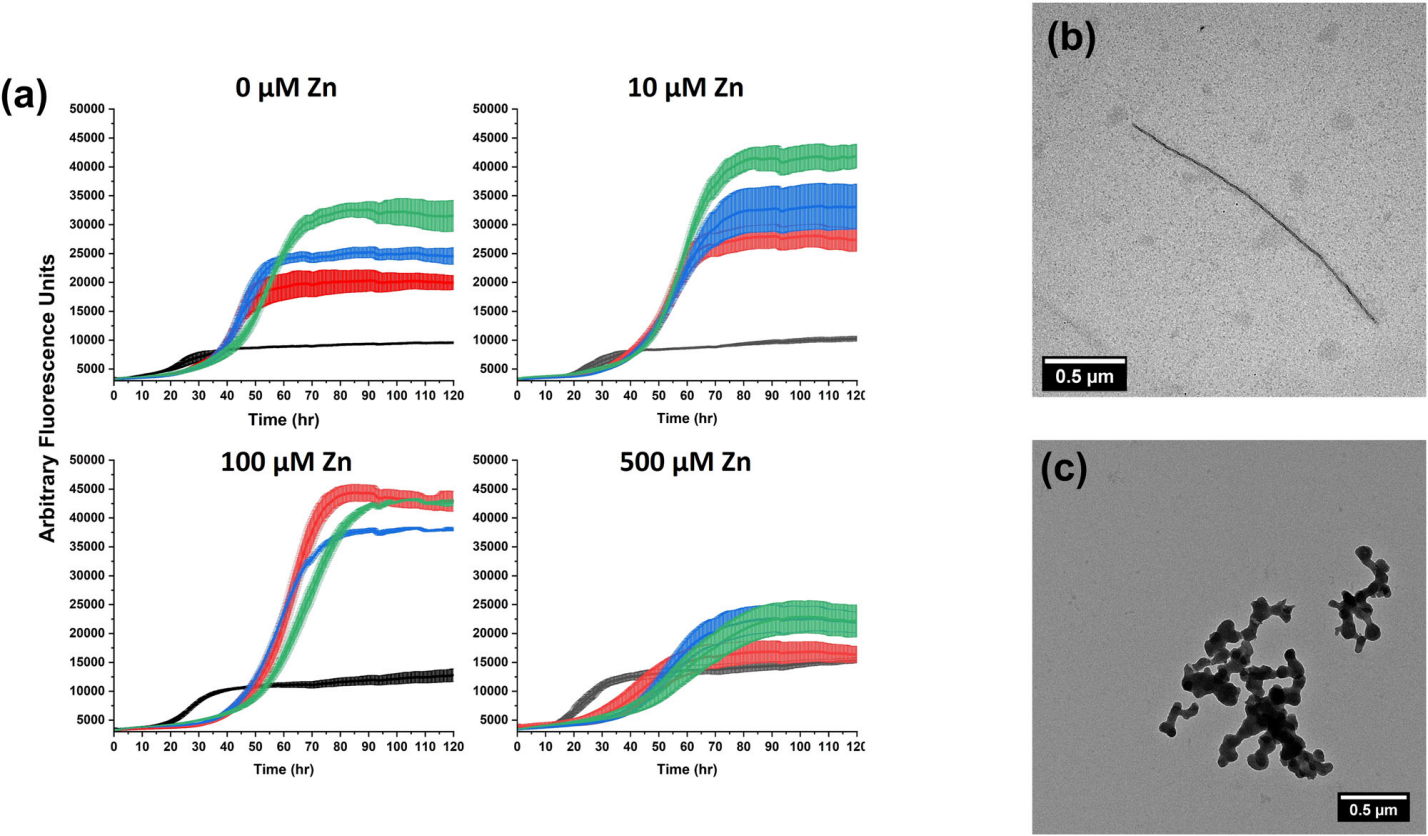
Aggregation of hIAPP in the presence of both zinc and insulin at pH 5.5. **a** ThT fluorescence assays were performed with 5 µM hIAPP, 0/2.5/5/10 µM insulin (black/red/blue/green), the noted concentrations of ZnCl_2_, 10 µM ThT, 10 mM NaAc, 100 mM NaCl, pH 5.5 samples. TEM micrographs were collected for samples with **b** 5 µM hIAPP, 0 µM insulin, and 5 mM ZnCl_2_ or **c** 5 µM hIAPP, 10 µM insulin, and 5 mM ZnCl_2_ after 96 hours sample incubation.

To model the physiological environment of hIAPP more accurately, we next investigated its aggregation in the presence of zinc and insulin together. We performed ThT fluorescence assays and TEM for mixed hIAPP-insulin-zinc samples in two pH conditions—pH 7.4 to model the extracellular space (Fig. 6) and pH 5.5 to reflect the acidity of the secretory granule (Fig. 7). At pH 7.4, each combination of zinc and insulin extended the lag phase of hIAPP aggregation compared to the control. However, zinc altered the dose-dependence of the inhibitory effect of insulin. Without zinc, increasing the concentration of insulin increased the length of the aggregation lag phase. While the behavior was similar with 10 µM zinc, the highest (500 µM) concentration of zinc caused *t*_1/2_ to be mostly independent of the concentration of insulin, albeit still extended relative to the without insulin control. In contrast, the intermediate concentration of zinc (100 µM) enhanced the inhibitory effect of insulin, and only a small increase in the ThT fluorescence was observed within 48 h with greater than equimolar insulin. At pH 5.5, the kinetics of hIAPP aggregation were less responsive to insulin concentration and mostly independent of zinc concentration. Again, the aggregation lag phase was most extended for the intermediate concentration of zinc, though less significantly than at the higher pH, while the high and low concentrations only caused marginal increases in the lag phase. These results were independently replicated in two labs (Figs. S7 and S8). Regardless of the pH, the TEM micrographs revealed fibrils for hIAPP and zinc samples without insulin and nonfibrillar aggregates for samples with hIAPP, insulin, and zinc. For comparison, another granule component that binds with insulin, C-peptide, had no effect on hIAPP aggregation except in the case of 5 µM hIAPP mixed with 25 µM insulin and 25 µM C-peptide (Fig. S9). In this condition, hIAPP aggregation was accelerated compared to 5 µM hIAPP mixed with 25 µM insulin and no C-peptide. C-peptide binds to insulin, so it is possible that in this case C-peptide competed with hIAPP for binding to insulin, thereby reducing the efficiency of insulin binding to hIAPP and inhibiting aggregation^74^.

**Fig. 8:**
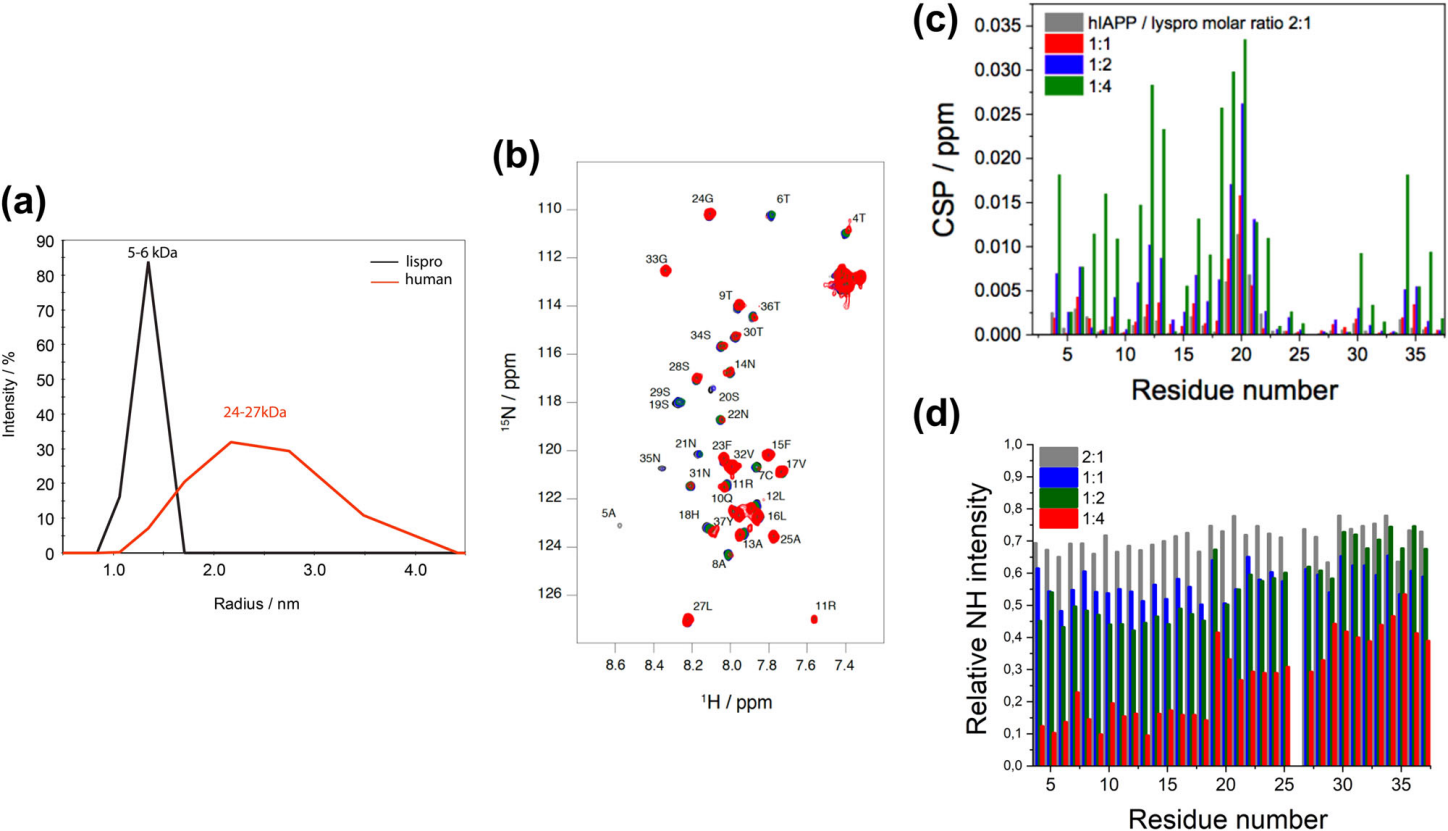
Monomeric insulin interacts more strongly with hIAPP than oligomeric insulin. **a** DLS size distributions of 170 µM human and lispro insulin show human insulin is oligomerized while lispro insulin is not. **b** Overlaid ^1^H-^15^N HSQC spectra of mixtures of 50 µM ^15^N-labeled hIAPP and unlabeled lispro insulin at pH 7.4 with 0 (black), 25 (gray), 50 (blue), 100 (green), or 200 (red) µM lispro insulin. hIAPP peak assignments are noted on the overlaid spectra. **c** CSPs and **d** peak intensities were calculated from the HSQC spectra. Sample conditions are the same as for Fig. 5b except for lispro insulin substituted for human insulin.

Given that zinc catalyzes the oligomerization of insulin into dimeric, tetrameric, and hexameric forms, we hypothesized that the complementary effects of zinc and insulin on hIAPP aggregation were due to interactions with oligomeric rather than monomeric insulin^56^. Unfortunately, we could not test this hypothesis directly because we were unable to measure the oligomeric state of insulin at the concentrations used in the ThT assays. We also could not increase the concentration of insulin because its oligomeric equilibrium is dependent on its concentration. To solve these problems, we used lispro insulin, which inverts B chain residues P28 and L29 and does not oligomerize, and compared its effect on hIAPP aggregation to that of wild-type (WT) human insulin (Fig. 8). DLS produced a sharp distribution of low molecular weight species for lispro insulin and a broad profile of larger species for WT human insulin (Fig. 8a), demonstrating that lispro insulin remained monomeric at a concentration that pushed WT insulin to form oligomers.

Our previous NMR experiments were conducted with higher insulin concentrations than the ThT assays, and the difference in the oligomeric state of insulin between the two experiments could account for the lack of interaction observed by NMR. We hypothesized that the interaction between hIAPP and the monomeric lispro insulin would be stronger and more observable by NMR; so we repeated the HSQC titration of ^15^N-labeled hIAPP with unlabeled lispro insulin (Fig. 8b). In fact, there was a much greater change in the HSQC spectrum of hIAPP when we titrated with lispro insulin. The largest CSPs were measured for hIAPP residues 4–20 (Fig. 8c), particularly residues L12, A13, H18, S19, and S20. A significant reduction in the signal intensity was also observed with more added insulin and was especially pronounced in residues 4–18 (Fig. 8d). However, even the largest CSPs were between 0.01 ppm and 0.03 ppm and were not saturated even with a four-fold excess of lispro insulin. Our results are consistent with previous work that reported a weak interaction between hIAPP and insulin, with a binding constant on the millimolar order of magnitude^42^.

### Insulin and zinc do not reduce cell toxicity associated with hIAPP

**Fig. 9:**
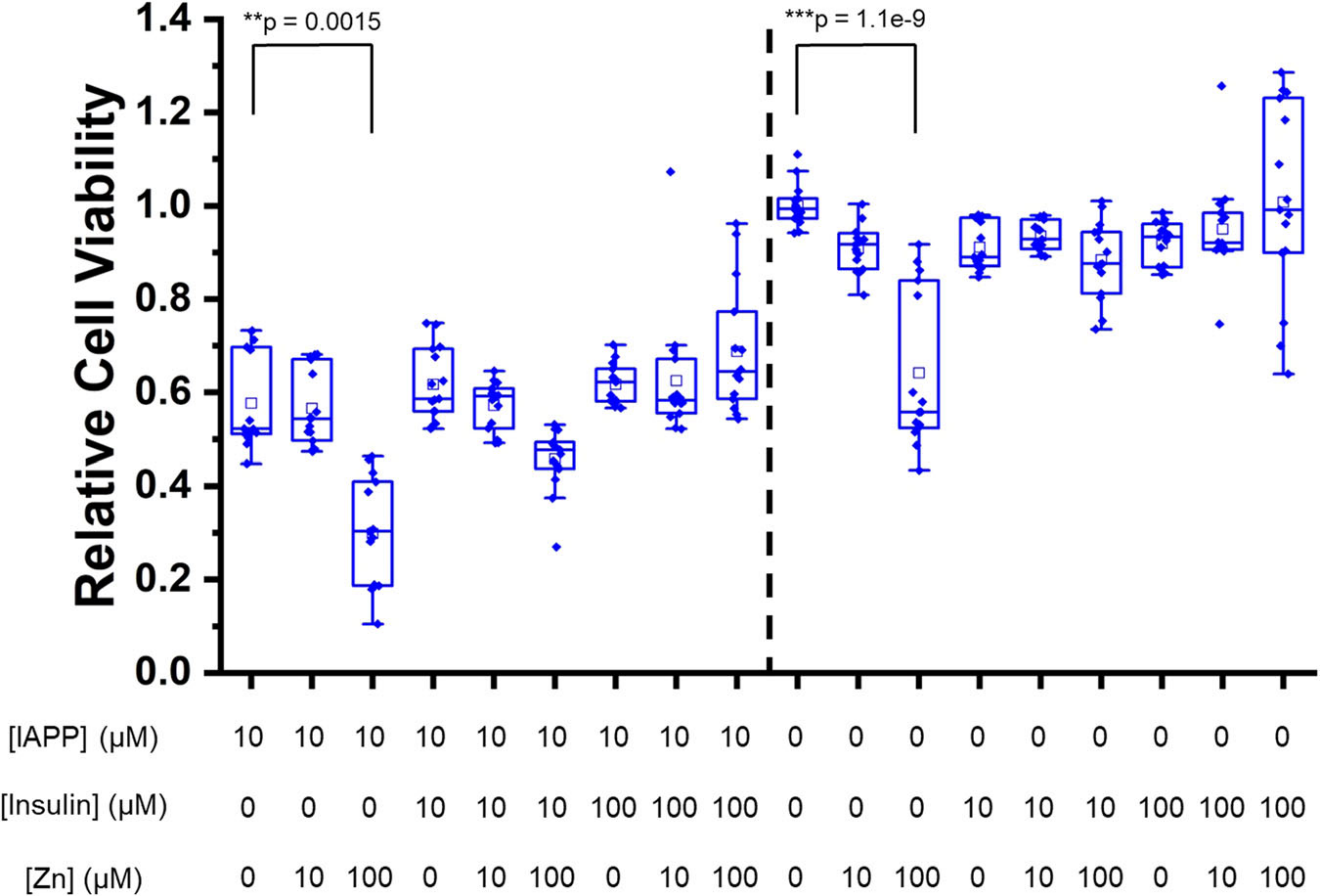
hIAPP-associated cytotoxicity is unaffected by zinc and insulin. MTT cell viability assays were performed after 48 hr incubation using RIN-5F cells in the presence of noted concentrations of hIAPP, insulin, and zinc. A dashed line separates the samples with and without hIAPP. For each sample condition, three independent experiments were performed with 5 replicates in each. The box-and-whisker plots display the sample median (solid line), mean (square), and scatter plot of raw data. Data were analyzed by the Kruskal-Wallis *H* test^80^. To determine statistically significant differences, samples in boxes 2–9 were compared to box 1, and boxes 11–18 were compared to box 10. Differences with *p* < 0.01 are shown.

To assess the significance of the interactions between hIAPP, zinc, and insulin on disease-associated cytotoxicity, we performed an MTT cell viability assay using a RIN-5F rat β-cell line (Fig. 9). hIAPP alone reduced the cell viability below 60% after 48 h of incubation. Adding insulin and zinc did not cause a statistically significant change in the cell viability, except for zinc added at tenfold excess. Insulin restored viability in this condition when equimolar with zinc, but only to the baseline of hIAPP alone (Fig. S10). Additionally, the toxic effect of high zinc concentration and restoration of cell viability by insulin was present in the absence of hIAPP, indicating that these arose from the toxicity of zinc alone and were not related to toxicity associated with hIAPP. Thus, though insulin and zinc clearly altered the aggregation of hIAPP, we cannot conclude from our data that they also reduced the cytotoxicity of hIAPP. However, we also note that we measured cytotoxicity at a single time point, and it is possible that while zinc and insulin did not decrease cell toxicity on the timescale of our experiment (48 hr), they might have delayed its onset by prolonging the formation of toxic hIAPP species.

## Discussion

hIAPP spontaneously aggregates within hours under in vitro conditions, but almost not at all in its native in vivo environment. Identifying the physiological cofactors or conditions underlying this difference could guide drug design efforts against amyloid formation associated with T2D. To this end, we characterized the combined effects of insulin, zinc, and low pH on hIAPP aggregation and toxicity. Our results fit squarely within the existing body of research on these systems. In agreement with previous work, we determined that insulin partially inhibited hIAPP aggregation in a concentration-dependent manner near neutral pH^35,36,47^. At acidic pH, insulin was a less effective inhibitor of hIAPP aggregation. A previous study reported a conflicting observation that insulin effectively inhibited hIAPP amyloid deposition under similar pH conditions but did not comment on kinetics^27^. The study was based on reduced Congo Red fluorescence in the presence of insulin, and it is possible that Congo Red would not have bound to nonfibrillar aggregates, which would be consistent with our results.

On that note, our detection of nonfibrillar aggregates formed from both hIAPP and insulin touches on a point of contention in the existing literature. Many early characterizations of hIAPP aggregation in the presence of insulin noted typical fibrillar aggregates were present in the TEM micrographs^36,43,47^. However, these reports all used either bovine insulin or an hIAPP:insulin ratio significantly greater than one. Experiments conducted under conditions more comparable to ours also produced nonfibrillar aggregates^37,41^. Amorphous aggregates of other proteins were demonstrated to inhibit the aggregation of Aβ40, likely by providing a non-specific hydrophobic surface area to sequester the peptide^75^. Insulin may act similarly on hIAPP; however, the increased end ThT fluorescence of hIAPP + insulin, relative to hIAPP fibrils, suggests that these nonfibrillar aggregates—unlike those observed for mixtures of amorphous aggregates and Aβ40—have a defined molecular structure. The elevated fluorescence intensity could arise from a greater aggregate yield by incorporating the insulin or from non-amyloid molecular structures such as what has been observed for beta-2 microglobulin^76^. More research is needed to explain this effect. That being said, we provided, to our knowledge, the first reports of biphasic ThT fluorescence kinetics, possibly corresponding to the growth of these aggregates and their potential molecular structure.

We also found that insulin prevented the self-association and structuring of hIAPP at the beginning of its aggregation pathway. Our CD data replicated previous work, and another NMR kinetics experiment similarly found the mixture of hIAPP and insulin to be stable, though the measurement only lasted 12 h compared to 48 h here^41,50,51^. This result also complements a finding from ion-mobility mass spectrometry that insulin pushes the equilibrium of hIAPP away from an aggregation-prone extended conformer and toward a compact conformer^48^. While it has been previously proposed that such results would arise from binding between hIAPP and insulin monomers competing with binding between two hIAPP monomers, we provided direct experimental evidence for insulin-preventing dimerization of hIAPP monomers. A previous cross-linking experiment produced seemingly contradictory results, observing the hIAPP homodimer even in the presence of insulin^44^. This could be explained by the cross-linking experiment artificially altering the oligomeric equilibrium of the sample or by inefficient ionization causing a small dimer population to be invisible in our MS data. The same logic could explain why the cross-linking experiment observed an hIAPP-insulin heterodimer while our MS experiment did not, despite MD studies predicting the heterodimer to be more energetically favorable^53,55^. Regardless, both sets of data agreed that insulin reduced the population of hIAPP homodimers. Moreover, our characterization of the nature of the binding between hIAPP and insulin was consistent with the weak interaction described previously, and in agreement with a molecular mapping study, we established residues 5–20 of hIAPP to be most involved in the binding^51^. While we do not have definitive evidence to evaluate models for the binding interaction, models relying on electrostatic interactions with residue H18 of hIAPP seem unlikely based on the strength of the interaction with insulin decreasing at acidic pH, where histidine would be protonated.

While predicted by a previous MD study from Nedumpully-Govindan et al., we provided the first direct experimental evidence that hIAPP interacts preferentially with monomeric insulin compared to oligomeric insulin^53^. As Nedumpully-Govindan et al. demonstrated, this effect arises due to insulin binding to hIAPP and other insulin molecules via the same interaction interface. Given that zinc promotes the oligomerization of insulin, this finding would account for our finding that zinc alters the inhibitory activity of insulin on hIAPP aggregation. Specifically, the dominant effect was that in the presence of zinc, the inhibition became independent of the insulin concentration. We could not quantify the oligomer distributions in our experiments, but it is possible that insulin has a “critical oligomer concentration”, which is lowered in the presence of zinc. Insulin would remain monomeric below this limit, and above it all excess insulin would exist as oligomers which do not interact with hIAPP. Concentrations above the critical concentration, would contain the same amount of monomeric insulin and thus inhibit hIAPP aggregation equally. Low pH having the same effect would account for similar behavior being observed at acidic pH. However, this is purely speculative and does not account for concentration dependence at intermediate zinc concentrations. Future research could clarify this issue.

In contrast to zinc, we found that C-peptide did not alter hIAPP aggregation in the presence of insulin. This disagrees with one previous study which reported an inhibitory effect of C-peptide. However, that study also reported a more modest effect below 10× excess C-peptide, as was used in our experiment^77^. Another study reported that C-peptide enhanced hIAPP fibrillation, except in the presence of Ca^2+^ when it had an inhibitory effect^27^. The authors of that report also observed that insulin and C-peptide in combination affected hIAPP aggregation like insulin alone, as we reported here for almost every combination of insulin and C-peptide. Zn^2+^ was found to be complex with hIAPP and C-peptide and reduce hIAPP cytotoxicity^49^. The complexation did not alter the kinetics of hIAPP aggregation^49^. It is difficult to reconcile the observations of these conflicting reports just in terms of experimental conditions. Inhibition of hIAPP aggregation by C-peptide was observed at pH 7.4 and promotion of aggregation at pH 5–6, while the buffer conditions used to investigate the hIAPP-C-peptide-Zn^2+^ complex are unclear^27,49,77^. However, we did not observe inhibition of hIAPP aggregation by C-peptide at pH 7.4. Regardless, hIAPP and C-peptide interact weakly, as measured by collision-induced dissociation mass spectrometry, suggesting that any effect C-peptide has on hIAPP amyloid formation is likely modest^74^.

In the context of the existing literature, our results advance the understanding of how the physiological environment of hIAPP protects it from toxic aggregation. Given that insulin and zinc either have little effect or an enhancing effect on hIAPP aggregation below neutral pH, it is most likely that within the secretory granule, acidic pH is a more important factor for discouraging amyloid formation. However, no combination of insulin, zinc, and pH suppressed hIAPP aggregation for the lifetime of a secretory granule. Thus, some other cofactors, such as lipids or protein chaperones, must also assist in preventing hIAPP aggregation in vivo. Future research might elucidate such a cofactor. Once hIAPP leaves the cell, zinc and especially insulin may act together to block aggregation of the transiently highly concentrated hIAPP prior to its dilution throughout the body. In this model, acidic pH, zinc, insulin, and other secretory granule contents only inhibit hIAPP aggregation for long enough that hIAPP can perform its physiological function after short-term storage, which would account for the lack of protection against cell toxicity seen in cell viability assays. Zinc and human insulin are thus clearly not promising drug candidates on their own. However, future research might consider mechanisms by which low pH, zinc, and insulin inhibit hIAPP aggregation—e.g., by affecting the charge state of hIAPP or by binding to the insulin interaction interface—to guide drug design.

## Materials and methods

### Materials

Unlabeled, amidated human IAPP with an intramolecular disulfide bond between residues 2 and 7 was purchased from Anaspec or Bachem AG (purity ≥95%), and Thioflavin-T was purchased from Cayman Chemicals. ^15^N-labeled human IAPP was produced as described previously^78^. All other chemicals, including recombinant human insulin and C-peptide, were obtained from Millipore Sigma. All hIAPP was pre-treated with HFIP and lyophilized prior to use.

### ThT fluorescence assay

Stocks of insulin and C-peptide were prepared by adding buffer (10 mM Tris, 100 mM NaCl, pH 7.4 or 10 mM NaAc, 100 mM NaCl, pH 5.5) and enough NaOH to dissolve. A Tris buffer was chosen over PBS due to the precipitation of zinc phosphate in samples containing zinc. The stock pH was then titrated back to the appropriate buffer pH with HCl. ZnCl_2_ stocks were diluted with buffer from 100 mM in pure H_2_O and pH was corrected back to 7.4 to account for the acidity of ZnCl_2_. All samples were mixed on ice to reach final concentrations of 5 µM hIAPP and 10 µM ThT in the appropriate buffer. Lyophilized hIAPP was dissolved in the buffer to 100 µM immediately prior to mixing in each sample and was always added last. Prior to placing in the microplate, all samples were centrifuged for 1 minute at 15,000 × *g* to remove large particles. For pH 7.4 samples, 100 µL of each sample was added in triplicate to 96-well, transparent round-bottom microplates (Fisher 12-565-500). For pH 5.5 samples and samples with C-peptide, 50 µL of each sample was added in triplicate to a 384-well, clear- and flat-bottomed, black-walled microplate (Greiner 07-000-892). Each kinetic experiment was preceded by 10 s of shaking and then performed under quiescent conditions. Fluorescence was measured using a BioTek Synergy 2 (insulin and zinc samples) or BMG Labtech FLUOstar Omega (C-peptide samples) microplate reader by exciting at 454 nm and reading emission at 480 nm every 8 minutes. The temperature was maintained at 25 °C for all experiments. Data is plotted as an average of 3 replicates per sample with error bars denoting one standard error. Amylofit was used to calculate the time to reach half-maximal fluorescence intensity (*t*_1/2_) for each raw data set, and *t*_1/2_ values are reported for each sample as the average of these *t*_1/2_ values, with error bars representing one standard deviation^79^.

### TEM

Aliquots (4.5 µL) of hIAPP (5 µM) were blotted for one minute on glow-discharged, carbon-coated, 200-mesh copper grids and dried on filter paper. The grids were then stained twice with 4% uranyl acetate for one minute and dried after each staining. TEM observation was performed with a FEI-CM 120 electron microscope, operated at 120 kV. Images were recorded with a US1900 GATAN CCD camera (Gatan, Pleasanton, CA, USA). At low magnification, ~25 squares were observed; at high magnification, around 10 squares were observed.

### Tandem liquid chromatography-mass spectrometry

Mixed and alone samples of hIAPP (5 µM) and insulin (5 µM) were prepared for a ThT fluorescence assay as described above, except for using a 10 mM Tris, 7 mM NaCl, pH 7.4 buffer. Aliquots were taken for analysis by LC-MS immediately after sample preparation and after the first fluorescence plateau (70 hr). Each LC-MS experiment was initiated by injecting 5 µL of sample onto a C18 column (Zorbax) with a particle size of 3.5 µm and dimensions of 2.1 mm × 50 mm. Elution was performed with a linear gradient of 5–100% 0.1% formic acid in acetonitrile against 0.1% formic acid in H_2_O over 14 minutes flowing at a rate of 0.8 mL/min. ThT eluted after 5.25 minutes, insulin after 5.60 minutes, and hIAPP after 5.99 minutes. Eluant from the column was analyzed by Q-TOF ESI mass spectrometry in positive ion mode. Both HPLC and mass spectrometry were performed using an Agilent Q-TOF HPLC-MS.

### CD spectroscopy

Samples were prepared as above with mixed or alone 25 µM hIAPP and 25 µM insulin. CD spectra were collected every hour at 25 °C using a Jasco CD-spectropolarimeter with averaging of 10 accumulations, 100 nm/minute scanning speed, 1 nm bandwidth, 0.5 nm data pitch, 1 s data integration, and 200 mdeg CD scale.

### ^1^H NMR

hIAPP samples were prepared with 50 µM hIAPP, 10 mM d_11_-tris, 100 mM NaCl, 10% D_2_O for locking, pH 7.4, and either 0 or 70 µM insulin. ^1^H NMR spectra were collected using a triple-resonance TXI probe with a 500 MHz Bruker spectrometer. Spectra were collected as an average of 1024 scans, acquired with a 10 µs 90° pulse and a 3 s recycle delay. Integrations were taken from 8.8–6.5 ppm and normalized relative to the integration of the spectrum at the first time point for the same sample.

### ^1^H-^15^N HSQC

Lyophilized hIAPP was dissolved directly into 50 mM phosphate buffer containing 10% D_2_O for locking. The samples were measured at 4 °C, employing Shigemi NMR tubes (Shigemi Inc., Allison Park, USA) and a Bruker Avance 600 MHz spectrometer, equipped with cryo-probe for solution-state NMR measurements. Backbone assignments were obtained from previously published data^78^. To avoid dilution of the hIAPP signal, hIAPP, and insulin mixed samples were prepared separately for each titration point from the same stock solutions.

### Dynamic light scattering

To probe the oligomerization of the insulin variants, we performed DLS experiments using a DynaPro NanoStar (Wyatt Technology). Both variants were measured as 100 µL of 1 mg/mL insulin in single-use cuvettes. The data was analyzed using the software package DYNAMICS V7.

### Cell toxicity

RIN-5F rat pancreatic beta cells (ATC CRL-2058, batch 61465080) were grown in RPMI-1640 medium with 10 mM glutamine, 10% FBS, and 100× Penicillin/Streptomycin at 37 °C and under 5% CO_2_. Cells were discarded after 25 passages. In a 96-well microplate, 100,000 cells were plated per well in 90 µL media and incubated for 24 h to let the cells adhere. Ten-fold concentrated stocks for each sample were prepared on ice immediately prior to adding them to cells. For each sample, 10 µL was added to cells in 5 separate wells. After an additional 48 h of incubation at 37 °C under 5% CO_2_, toxicity was assessed using the MTT cell viability assay (Promega PAG4000), according to the manufacturer’s protocol. MTT dye solution (15 µL) was added to each sample. The cells were incubated for another 4 h, and then 100 µL of stop solution was added to each well. Absorbance was measured at 570 nm and 700 nm. The difference between A_570_ and A_700_ was calculated for each replicate of each sample. This difference from a sample with 1% SDS was subtracted from the absorbance values for each sample replicate, and the absorbance values were normalized relative to the average absorbance of the buffer control. Toxicity was independently measured three times in this way. Cell viability was reported as an average of 15 replicates for each sample. Differences between sample conditions were compared by the Kruskal–Wallis *H* test, and statistical significance was determined as *p* < 0.01^80^.

### Supplementary information

Supplemental Information

## Data Availability

All data supporting our conclusions are provided in the main text and supporting information. The raw data and the numerical source data behind graphs can be found in the Open Science Framework (Project Title: McCalpin et al. *Commun. Biol*. 2024—Raw Data, https://osf.io/rbcfd/?view_only=b3fc314c033b446eb5338d30b16433f4).
