# Supplementary material for: Zinc and pH modulate the ability of insulin to inhibit aggregation of islet amyloid polypeptide
Source: Commun Biol. 2024 Jun 27;7:776. doi: 10.1038/s42003-024-06388-y (PMC11211420; doi:10.1038/s42003-024-06388-y)
Supplement: Supplementary file 1 — Supplemental Information [file 42003_2024_6388_MOESM1_ESM.pdf]

Zinc and pH Modulate the Ability of Insulin to Inhibit Aggregation of Islet Amyloid Polypeptide

AUTHOR NAMES

Samuel D. McCalpin[1,2], Lucie Khemtemourian[3], Saba Suladze[4,5], Magdalena I. Ivanova[1,6,7], Bernd Reif[4,5], Ayyalusamy Ramamoorthy[1,2,6,7,8,9]*

AUTHOR ADDRESS

[1]Biophysics Program, University of Michigan, Arbor, MI 48109, USA

[2]Department of Chemistry, University of Michigan, Arbor, MI 48109, USA

[3]Institute of Chemistry and Biology of Membranes and Nanoobjects (CBMN), CNRS - UMR 5248, Institut Polytechnique Bordeaux, University of Bordeaux, 33600, Pessac, France

[4]Bayerisches NMR Zentrum (BNMRZ) at the Department of Biosciences, School of Natural Sciences, Technische Universität München, Munich, Germany

[5]Helmholtz-Zentrum München (HMGU), Deutsches Forschungszentrum für Gesundheit und Umwelt, Institute of Structural Biology (STB), Ingolstädter Landstr. 1, 85764 Neuherberg, Germany

[6]Department of Neurology, University of Michigan, Arbor, MI 48109, USA

[7]Michigan Neuroscience Institute, University of Michigan, Arbor, MI 48109, USA

[8]Biomedical Engineering, Macromolecular Science and Engineering, University of Michigan, Ann Arbor, MI 48109, USA

[9]National High Magnetic Field Laboratory, Department of Chemical and Biomedical Engineering, Institute of Molecular Biophysics, Neuroscience, Florida State University, Tallahassee, FL 32310, USA.

AUTHOR INFORMATION

Corresponding Author

*aramamoorthy@fsu.edu

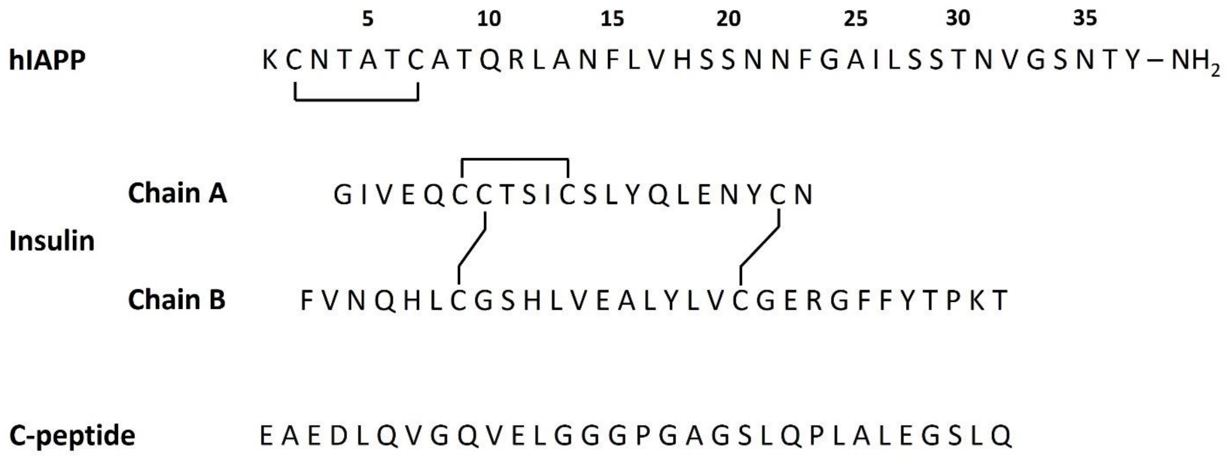

Figure S1. Amino acid sequences of human IAPP, human insulin, and human C-peptide. Disulfide bonds are indicated where present and the amidated C-terminus of hIAPP is noted.

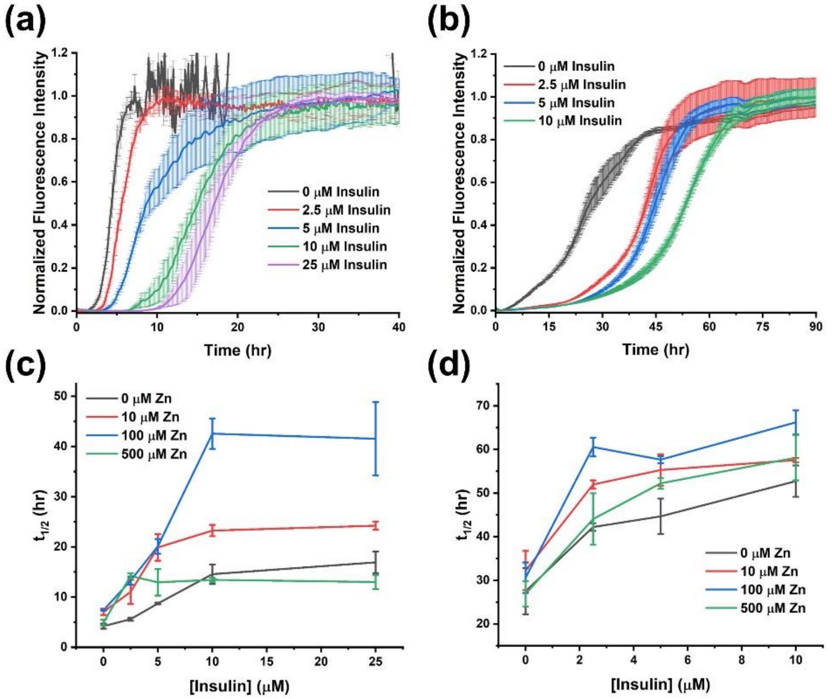

Figure S2. Normalized ThT fluorescence data of hIAPP and insulin and calculated half-times. ThT fluorescence assays were performed with 5 μM IAPP, 10 μM ThT, and the noted concentrations of insulin and $ZnCl_2$ in either **(a)** 10 mM Tris, 100 mM NaCl, pH 7.4 or **(b)** 10 mM NaAc, 100 mM NaCl, pH 5.5. For samples at **(c)** pH 7.4 and **(d)** pH 5.5, the time taken to reach half the maximum fluorescence intensity was calculated and plotted versus the concentrations of insulin and zinc.

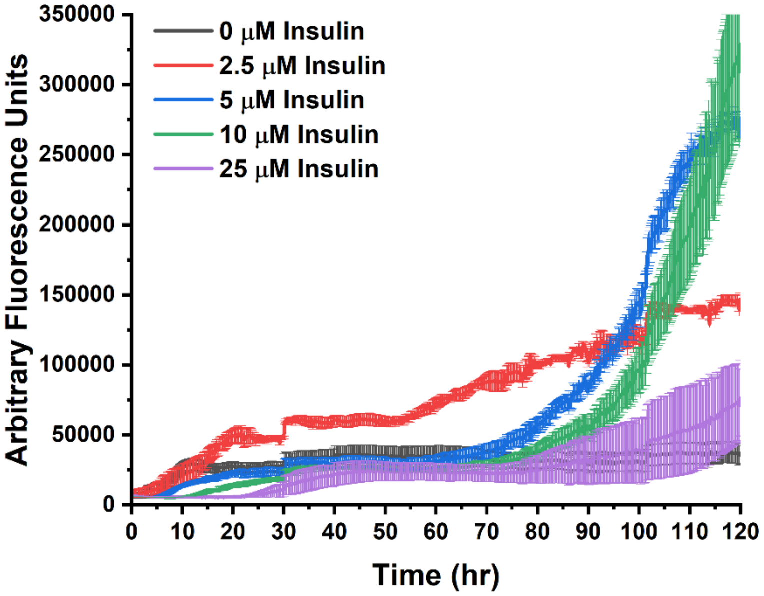

Figure S3. Long-time ThT fluorescence kinetics of IAPP + insulin reveal a second increase in the ThT fluorescence intensity. Samples contained 5 μM IAPP, the noted concentrations of insulin, 10 μM ThT, 10 mM Tris, 100 mM NaCl, pH 7.4.

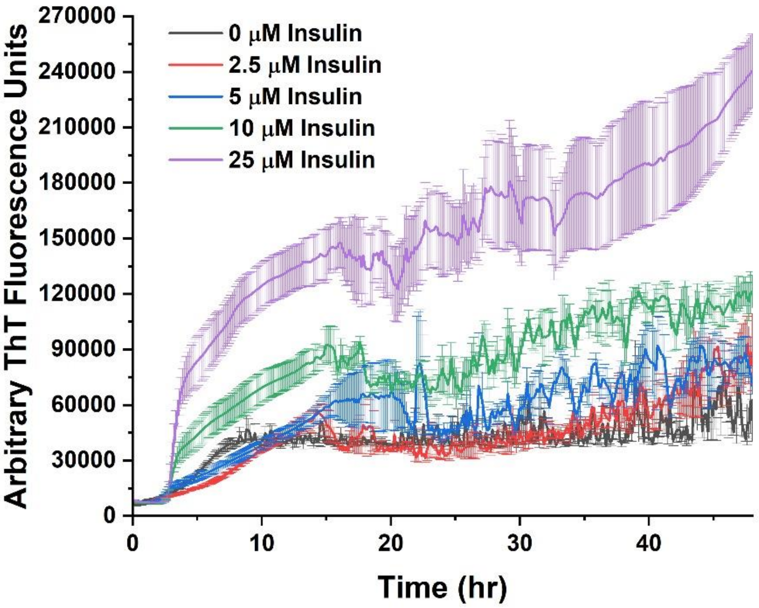

Figure S4. MES accelerates IAPP aggregation. ThT fluorescence assay of 5 μM IAPP, 10 μM ThT, 20 mM MES, 100 mM NaCl, pH 5.5, and the noted concentrations of insulin. Note that the lag times are significantly shorter than samples that are identical except for using sodium acetate as the buffer in place of MES (**Figure 1**).

(a) 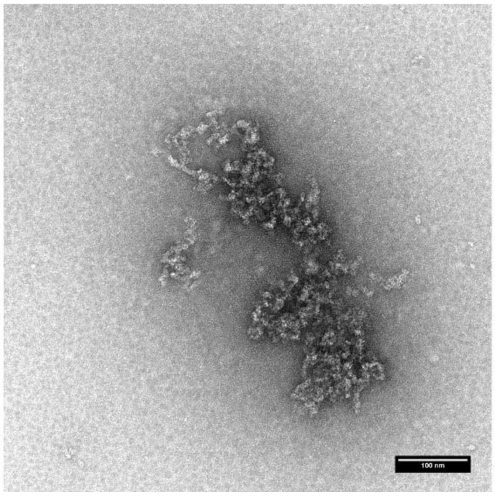

(b) 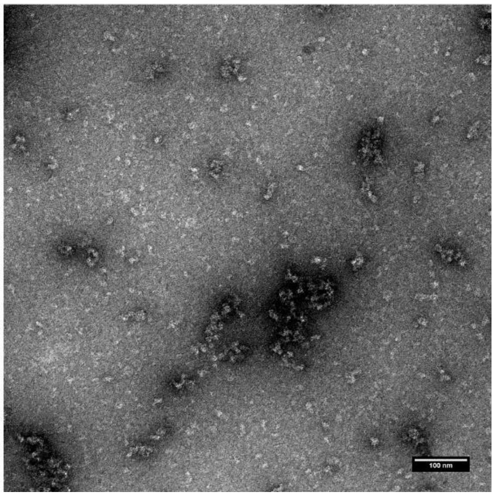

Figure S5. Representative TEM micrographs of 5 μM insulin incubated for 96 hr in **(a)** 10 mM Tris, 100 mM NaCl, pH 7.4 or **(b)** 10 mM MES, 100 mM NaCl, pH 5.5.

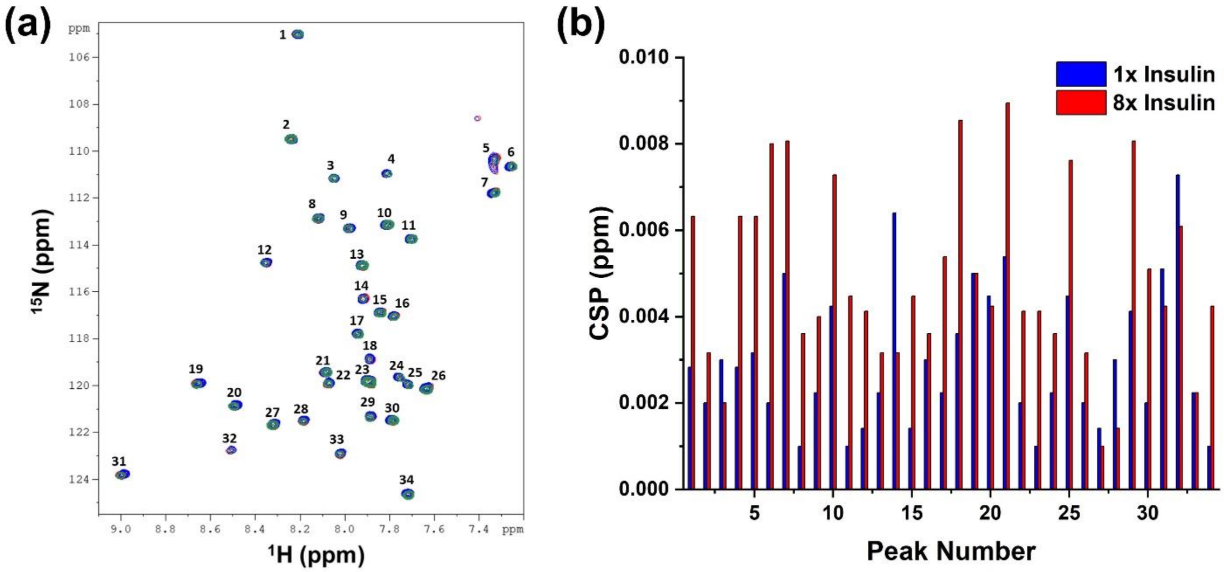

Figure S6. ^{1}H-^{15}N HSQC NMR Titration of IAPP with human insulin in a helix-inducing solvent. **(a)** Overlaid ^{1}H-^{15}N HSQC NMR spectra were plotted for 50 µM ^{15}N-labeled IAPP with 0 (red), 50 (blue), or 400 (green) µM insulin in a 30% HFIP buffer (70% water, 30% HFIP, 10 mM Tris, 100 mM NaCl, pH 7.4). Resonances were arbitrarily labeled, and **(b)** CSPs were calculated for each resonance. Note that the peak labels are not IAPP residue assignments. The CSPs were too small to definitively determine as arising from an interaction between IAPP and insulin rather than artifacts of small differences in sample composition (e.g., pH, salt concentration, and HFIP content).

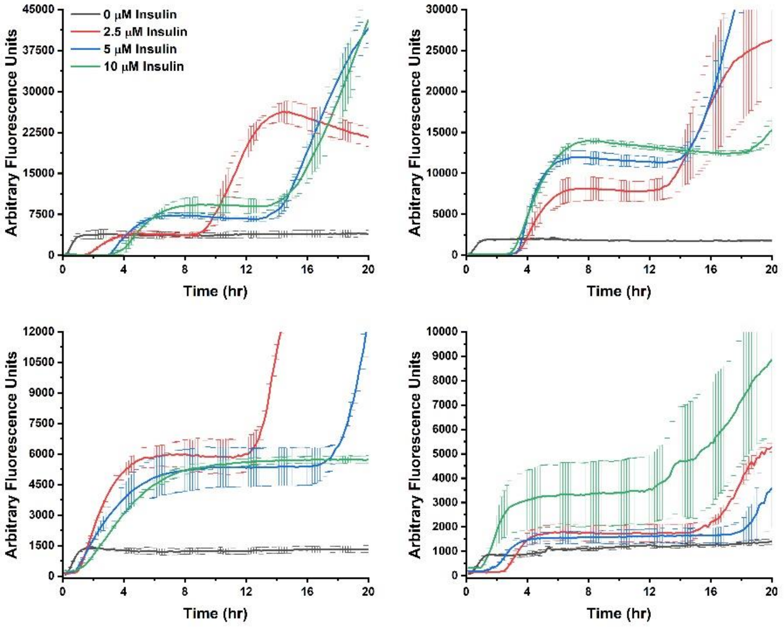

Figure S7. Replication of aggregation of IAPP in the presence of both zinc and insulin at pH 7.4. ThT fluorescence assays were performed independently in a separate lab (see Methods) with identical sample conditions as the assays shown in **Figure 6**.

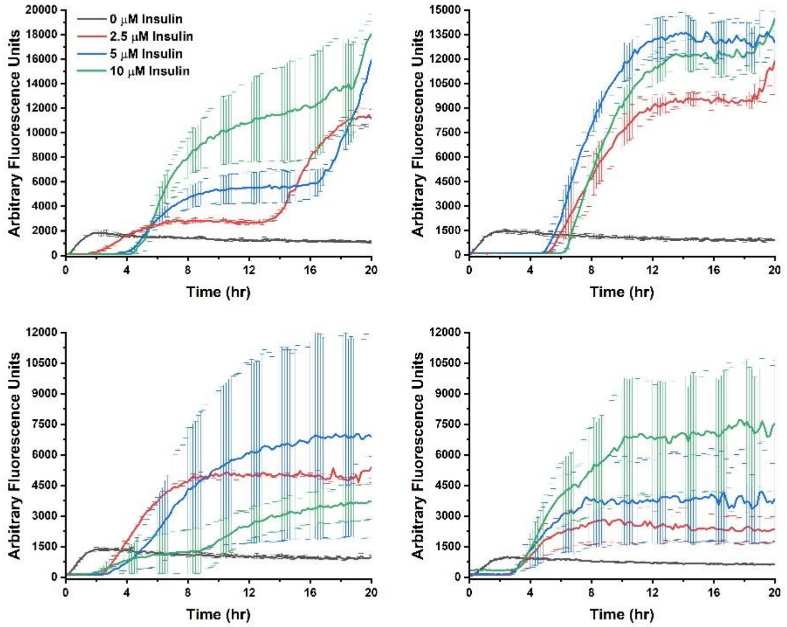

Figure S8. Replication of aggregation of IAPP in the presence of both zinc and insulin at pH 5.5. ThT fluorescence assays were performed independently in another lab (see Methods) with identical sample conditions as the assays shown in **Figure 7**, except for 10 mM MES used in place of 10 mM NaAc.

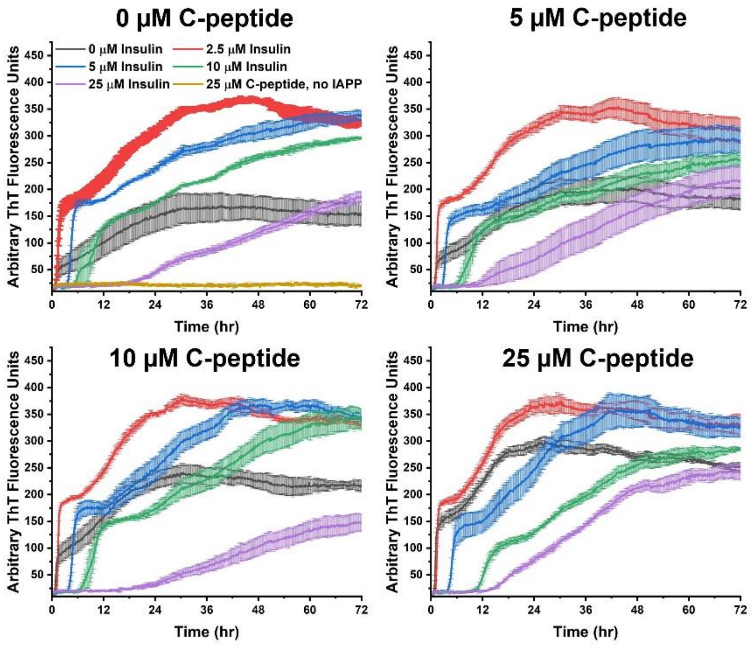

Figure S9. Effect of C-peptide on insulin's inhibition of hIAPP aggregation. ThT fluorescence assays were performed on samples with 5 μM hIAPP, 0/2.5/5/10/25 μM insulin (black/red/blue/green/purple), the noted concentrations of ZnCl2, 10 μM ThT, 10 mM Tris, 100 mM NaCl, and pH 7.4.

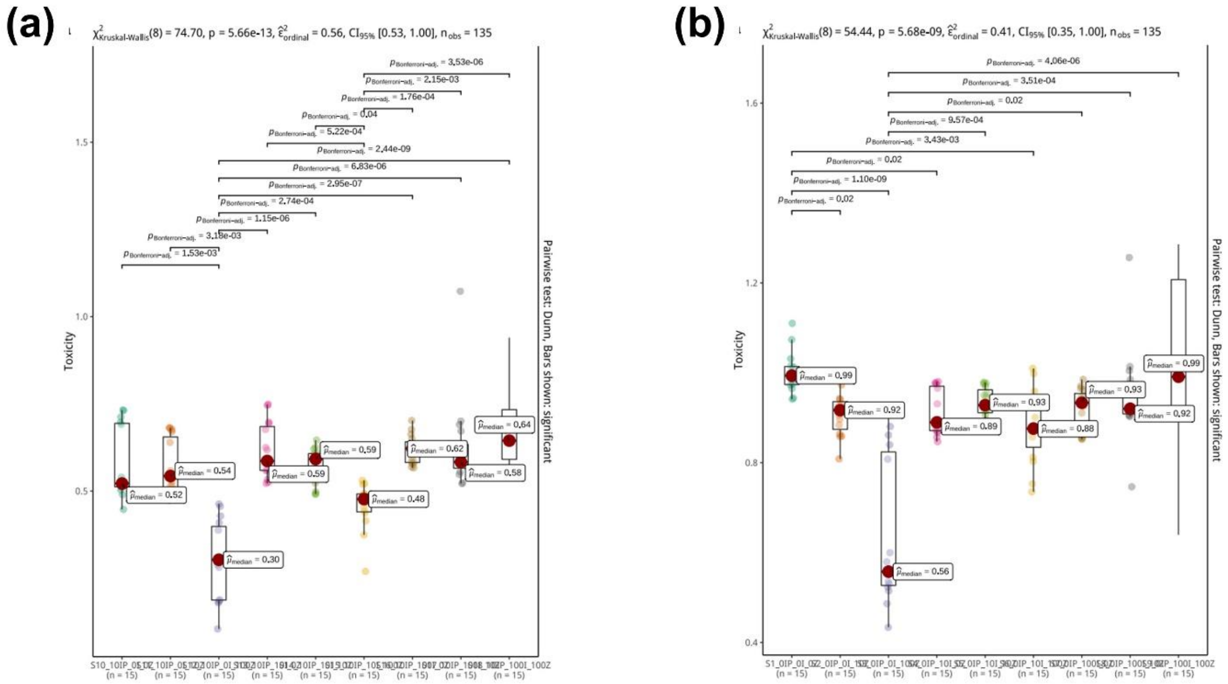

Figure S10. Full statistics of MTT cell viability assays of RIN-5F cells with **(a)** 10 µM IAPP or **(b)** 0 µM IAPP. *Data were analyzed by* a Kruskal-Wallis H test and p values are noted for each sample pair. Samples are ordered as in **Figure 9**.[1]

References

(1) Jiang, W.; Chen, H.; Yang, L.; Pan, X. moreThanANOVA: A User-Friendly Shiny/R Application for Exploring and Comparing Data with Interactive Visualization. *PLOS ONE* **2022**, *17* (7), e0271185. https://doi.org/10.1371/journal.pone.0271185.
